# Multi-stage decimation with hybrid CIC-polyphase filtering for IoT gateway sample rate conversion

**DOI:** 10.1038/s41598-025-31617-7

**Published:** 2025-12-23

**Authors:** Swetha Pinjerla, Surampudi Srinivasa Rao, P Chandrasekhar Reddy

**Affiliations:** 1 Department of ECE, Jawaharlal Nehru Technological University, Hyderabad, India; 2 Department of ECE, Muffakham Jah College of Engineering and Technology, Hyderabad, India

**Keywords:** Cascaded integrator-comb, Polyphase, Decimation filters, Virtex, FPGA, Energy science and technology, Engineering, Mathematics and computing

## Abstract

In real-time digital signal processing, the multirate transformation is a commonly used technique for interpolation and decimation. Comb-based decimation filters are used in wireless applications because of their minimal complexity and effective alias suppression. One symmetrical Finite Impulse Response (FIR) filter that may be used as a decimation filter is the cascaded integrator-comb (CIC) filter. Compared to other decimation filters, the CIC filter operates faster and uses less hardware because it does not require multipliers. Efficient digital filtering is critical in modern wireless communication systems, where real-time processing and resource optimization are essential. This paper presents a multi-stage hybrid polyphase CIC filter architecture implemented on a Xilinx Virtex-4 Field-Programmable Gate Array (FPGA) to enhance signal processing performance. The proposed method integrates a polyphase CIC filter with an FIR compensation filter, addressing the passband droop and improving frequency response. By leveraging polyphase decomposition and pipeline optimization, the design reduces computational complexity while maintaining high accuracy. The FPGA-based implementation ensures low latency, high throughput, and efficient hardware utilization, making it ideal for high-speed wireless applications. Experimental results demonstrate that the proposed filter achieves superior filtering performance compared to conventional CIC filters, offering a robust solution for real-time digital signal processing. The proposed method achieves substantial hardware savings, utilizing only 289 Slice Registers, 25 LUTs, 346 Flip-Flops, 63 BRAMs, and 76 DSPs, compared to the highest values from prior works, which consumed up to 628 Registers, 442 LUTs, 654 BRAMs, and 364 DSPs. This represents a reduction of up to 54% in Slice Registers, 94% in LUTs, and 88% in DSPs, highlighting the efficiency of the proposed architecture for SDR-based IoT gateways.

**Supplementary Information:**

The online version contains supplementary material available at 10.1038/s41598-025-31617-7.

## Introduction

Digital down converters (DDCs) are essential for imaging, sensing, and communication. In order to convert an intermediate-frequency (IF) signal into a complex low-frequency signal for further processing, it employs channel filtering and a sample rate changer^1^. A high-performance DDC architecture must be developed in order to handle high sampling rate signals, which are often produced by an analog-to-digital converter (ADC), usually an ADC12D1800, due to the fast advancement of technology. The development of a DDC using modern FPGA technology offers greater flexibility and lower power consumption compared with application-specific integrated circuits (ASICs)^2^. The process of changing a data stream’s sampling frequency is known as sample-rate conversion (SRC). SRC is an essential component of many modern applications, including digital mixing consoles, audio workstations, and various software-defined processing systems. Most high-quality SRC systems upsample the input to a very high rate and then downsample it to the desired output rate using digital filtering techniques^3^.

In communication infrastructures, digital filters have also become a powerful choice for eliminating noise, modifying the spectrum, and reducing inter symbol interference. Because of its accurate repeatability, which enables design engineers to attain performance levels that are hard to attain with analog filters, these filters have gained popularity. The CIC filter is a digital filter well suited for multiplier-less implementations. There are several uses for this kind of filter in the low-cost construction of decimators and interpolators. Nevertheless, pass-band droop is an issue that may be resolved using compensating methods. Another popular family of digital filters for arbitrary sample rate conversions and fractionally delaying samples are the Farrow filters. They are very effective for digital filtering and feature a poly-phase structure. Furthermore, FPGA have emerged as a very affordable way to offload computationally demanding digital signal processing algorithms in order to enhance system performance^4^. In modern communication networks, the DDC is an essential component. DDC’s basic idea is to convert high sample rates to low sample rates while maintaining a reasonable gain. Wideband and narrowband DDC are the two primary categories of DDC. Wideband DDCs are appropriate for 4G WiMAX and satellite applications because of their bandwidth exceeding 1 MHz. Narrowband DDCs, on the other hand, are used in commercial broadcast systems and frequency division multiplexed (FDM) systems, and their bandwidth is less than 1 MHz. Creating a reconfigurable, high-performance Software Defined Radio (SDR) system is quite desired in the current industry^5^.

It is necessary to point out that this work does not purport theoretical novelty in CIC structures or polyphase filtering, both of which have long histories in multirate signal processing. What this paper brings to the fore is rather the optimization of architectures and the multi-protocol design that is specialized to low-power IoT receivers. In particular, we present a hybrid CIC-polyphase decimation architecture that enables LoRa, SigFox, and NB-IoT to be used on the same reconfigurable hardware platform, as well as a rational approach to the choice of CIC parameters (R, N, D) and FIR compensation order depending on the DRC specifications. These application-controlled optimizations make it possible to achieve large resource utilization and power savings as well as address alias suppression and passband fidelity criteria. The proposed Multi-Stage Hybrid Polyphase CIC Filter enhances digital filtering in wireless communication systems by integrating polyphase decomposition with an FIR compensation filter, reducing passband droop and computational complexity. Implemented on a Xilinx Virtex-4 FPGA, it optimizes hardware resources while ensuring low latency, high throughput, and power efficiency. The use of Block RAM and DSP slices improves memory and arithmetic operations, making it ideal for real-time signal processing.

The paper is structured as follows: Section II reviews existing CIC filter architectures and their limitations. Section III presents the proposed Multi-Stage Hybrid Polyphase CIC Filter, detailing its design methodology and FPGA implementation. Section IV discusses the experimental setup, ASIC performance metrics, resource utilization analysis and comparative results, demonstrating the efficiency of the proposed method. Finally, Section V concludes the paper with key findings and future research directions.

## Literature survey

Over the last ten years, a number of research publications on area and power optimization in DDC systems employing FPGA have been published.

The CIC Decimation Filter Using Redundant Number System was suggested by Ajeet et al. For modern digital signal processing (DSP) applications, power, speed, and hardware resource optimization are the primary design goals. Digital systems use the redundant number system to lower computational burden; circuit-level architectural modifications may increase this efficiency even further. In order to boost attenuation in the folding band and decrease passband droop, the signed digit-based FIR filter is suggested as a compensating filter. When compared to the current structure, the suggested compensation filter with CIC filter design results in a 17.27% decrease in passband droop and a 12.29% improvement in attenuation in the folding band. Furthermore, compared to the current architecture, the simulation results indicate a 10.44% reduction in the number of LUTs used^6^.

Architectures for decimation filter networks of digital receivers that use simplified logic and may receive various communication standard signals were suggested by Latha et al. Additionally, multi-stage multi-rate filter designs with decreased Very Large Scale Integrated (VLSI) cost functions are designed, simulated, and implemented. To address the need of receiving multistrand receiver signals, the Multi-Standard Decimation Filter (MSDF) structure is presented in the first multi-stage design. CIC filters are used in the first phases of the MSDF architecture, which is intended for GSM and WiMAX wireless communication requirements. To satisfy the multi-standard criteria, the second design uses polynomial CIC filters to construct a modified MSDF structure. The third architecture focuses on the implementation principles and design parameters of a polyphase CIC-based decimation filter^7^.

In order to satisfy the requirements of a Global System for Mobile (GSM) receiver, Debars et al. suggested designing and implementing a reconfigurable DDC that can handle input bandwidth ranging from around 70 MHz to 270 kHz. Multi-rate decimation filters and the Coordinate rotation digital computer (CORDIC) processor make up the suggested design. The design’s greatest spurious-free dynamic range (SFDR) was attained by using the CORDIC processor. Additionally, using a multi-rate decimation filter enhances the DDC design’s performance while requiring little hardware resources. Using a Xilinx Kintex-7 FPGA board, the suggested DDC was created and evaluated. The benefits of adopting this adaptable DDC include the ability to generate a certain output. According to experimental findings, the suggested DDC operates at a high processing speed and in the best possible area to provide a financially viable solution for mobile applications^8^.

A non-recursive CIC decimation filter’s polyphase segmentation is proposed by Abinaya et al. using various parallel prefix adders. Software-defined radio applications may benefit from the effective sample rate conversion technique known as polyphase decomposition. Compared to recursive CIC filters, the novel polyphase-based non-recursive CIC filter offers a unique design that provides faster processing rates and lower power consumption. This short examines the suggested non-recursive CIC and its polyphase architecture using Xilinx 14.7 on a Kintex7 FPGA. Also, MATLAB has been used to illustrate the magnitude response. Compared to the current multiplier less filter, the proposed polyphase multiplier less CIC filter with a hybrid PPF CSL adder reduces power consumption. A special polynomial function has been developed to improve the magnitude characteristic of the suggested filter, reducing stopband ripple by 73.41% and passband droop by 34.27% when compared to the current filter^9^.

A unique multistage CIC filter, which is essential for software-defined radio (SDR) applications, was suggested by Abinaya et al. Different sample rates are required by wireless networking standards to operate the baseband signal. Consequently, one of the most important SDR techniques is SRC. Therefore, a particular kind of linear-phase FIR filter that may be used as a decimation filter for SRC operation is the CIC filter. Compared to previous decimation filters, this one takes less space because of its multiplier less construction and minimal passband droop. This short examines the basic construction of the CIC filter using a variety of parallel prefix adders and provides examples of the important parameters of the CIC filter for each adder. Using Xilinx ISE 14.7, the suggested CIC filter with multiple parallel prefix adders was created in Verilog HDL and put into practice on a Kintex7 FPGA. In terms of size, speed, and power consumption, the schemed CIC filter design is contrasted with the conventional CIC filter. According to the findings produced on the Kintex7 FPGA, the CIC filter with Brent Kung adder performs better than the conventional CIC filter and other CIC filters employing other parallel prefix adders^10^.

## Multi-stage decimation with hybrid CIC-polyphase filtering for IOT gateway sample rate conversion

**Fig. 1:**
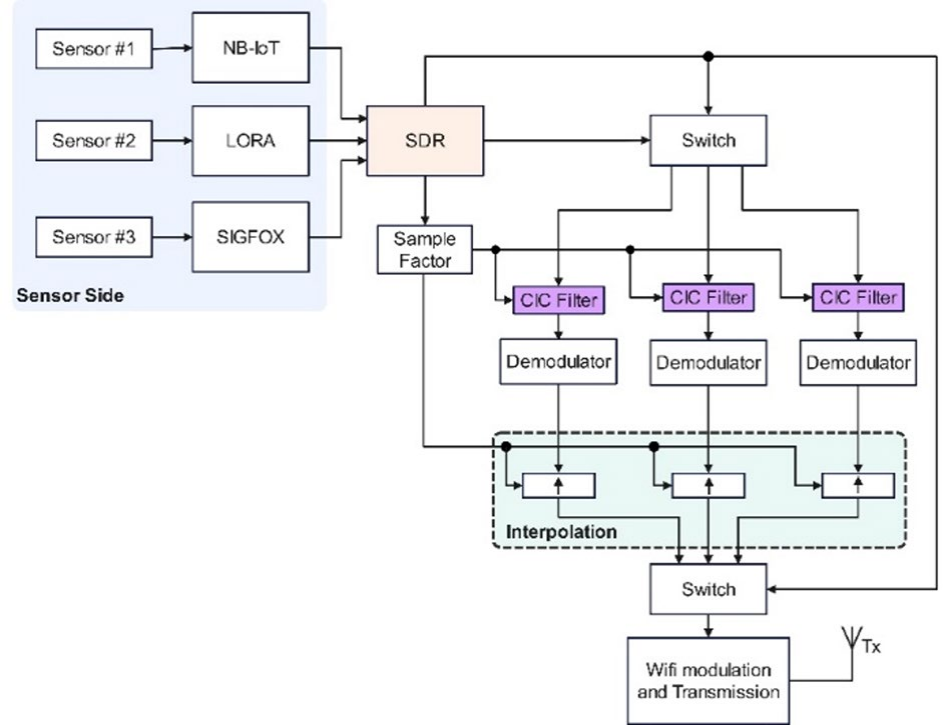
Block diagram of SDR based IoT interface model.

The mathematical formulation in this section follows the standard CIC filter model; our contribution does not lie in altering these classical equations. Instead, the novelty of our work is in the systematic methodology used to determine the optimal CIC structure—specifically the number of stages (N), decimation factor (R), and differential delay (D)—together with the order of the FIR compensation filter, all derived from protocol-specific DRC specifications. Traditional CIC design does not address the multi-protocol constraints of IoT receivers, where LoRa, SigFox, and NB-IoT exhibit significantly different bandwidths, symbol rates, and alias-suppression requirements. In our approach, R is selected based on the target output sampling rate for each protocol, while N and D are chosen to ensure that CIC passband droop and stopband attenuation remain within acceptable limits. A frequency-domain FIR compensation filter is then designed to correct residual distortion and enhance alias rejection, with its order determined by the required passband flatness and the roll-off characteristics of the chosen CIC configuration. This coordinated selection of CIC parameters and FIR equalizer order minimizes passband droop, controls aliasing, and efficiently utilizes FPGA resources. Overall, the proposed hybrid CIC–polyphase FIR architecture is optimized for hardware reuse and low-power operation across all three protocols. In contemporary wireless communication systems, where signals must be processed at many rates to guarantee smooth data transmission and reception, sample rate conversion, or SRC, is essential. Applications like digital receivers, multirate signal processing systems, and Software-Defined Radio (SDR) all depend on SRC^11–13^. Traditional SRC methods are ineffective for real-time FPGA implementations because they depend on Finite Impulse Response (FIR) filters, which need a lot of processing power. Because of their multiplier-less design, CIC filters provide an effective solution to this problem and are thus a good fit for hardware-based sample rate conversion. CIC filters enable decimation and interpolation while minimizing hardware complexity. Their structure consists of cascaded integrator and comb stages, which allow efficient filtering operations without the need for multipliers, reducing FPGA resource utilization^14^. Figure 1 illustrates an SDR-based IoT gateway architecture designed to support multiple sensor-side communication protocols, specifically NB-IoT, LoRa, and SigFox. Each sensor transmits data using one of these protocols, which is then received and digitized by the SDR module. The SDR forwards the digitized signals to a configurable switch and a sample factor control block, which manages data routing and rate adjustments. Each signal path includes a dedicated CIC filter to perform decimation, followed by a demodulator to extract the baseband data. The baseband signals are then passed through an interpolation block to upsample them uniformly before being routed via another switch. Finally, the unified data is modulated onto a Wi-Fi signal and transmitted to the cloud or access point for further processing or storage.

### Mathematical model for multi-stage decimation with hybrid CIC-polyphase structure

#### Multi-stage decimation model

If the total decimation factor is $\:R$, instead of a single-stage decimation, we factorize it into multiple smaller decimations:

1 $$\:R={R}_{1}\times\:{R}_{2}\times\:\dots\:.\times\:{R}_{N}$$

where $\:{R}_{i}\:$are the individual stage decimation factors?

#### CIC transfer function (H(z))

A CIC filter consists of integrators (accumulators) and comb sections. The general transfer function is:

2 $$\:H\left(z\right)={\left[\frac{1-{Z}^{-RM}}{1-{Z}^{-1}}\right]}^{N}$$

Where $\:R$ = decimation factor, $\:M$ = differential delay (default M = 1, but optimized for M > 1), N = number of integrator-comb stages. For multi-stage decimation, each stage $\:\boldsymbol{i}$ has its own transfer function:

3 $$\:{H}_{i}\left(z\right)={\left[\frac{1-{Z}^{-{R}_{i}M}}{1-{Z}^{-1}}\right]}^{{N}_{i}}$$

The overall CIC system is then:

4 $$\:{H}_{CIC}\left(z\right)={H}_{1}\left(z\right).{H}_{2}\left(z\right)\dots\:..{H}_{N}\left(z\right)$$

This reduces the hardware cost while maintaining the desired decimation.

#### Polyphase decomposition and computational optimization

Since CIC filtering involves high-rate data, we apply polyphase decomposition to optimize processing. The polyphase representation of a CIC filter with R-stage decimation is:

5 $$\:H\left(z\right)=\sum\:_{k=0}^{\mathbf{R}-1\text{}}{z}^{-k}{P}_{k}\left({z}^{R}\right)$$

where $\:{P}_{k}\left({z}^{\:}\right)$ are the polyphase components. Instead of computing every output sample, we only compute one of R polyphase components per clock cycle. This reduces the number of operations by R times, minimizing power consumption.

6 $$\:{H}_{CIC-polyphase}\left(z\right){H}_{i}\left(z\right)={\left[\frac{1-{\boldsymbol{Z}}^{-{\boldsymbol{R}}_{1}\boldsymbol{M}}}{1-{\boldsymbol{Z}}^{-1}}\right]}^{{N}_{1}}\times\:{H}_{i}\left(z\right)={\left[\frac{1-{\boldsymbol{Z}}^{-{\boldsymbol{R}}_{2}\boldsymbol{M}}}{1-{\boldsymbol{Z}}^{-1}}\right]}^{{N}_{2}}$$

With polyphase optimization

7 $$\:{H}_{\mathrm{C}\mathrm{I}\mathrm{C}-\mathrm{P}\mathrm{o}\mathrm{l}\mathrm{y}\mathrm{p}\mathrm{h}\mathrm{a}\mathrm{s}\mathrm{e}\text{}}\left(z\right)=\sum\:_{k=0}^{\mathbf{R}-1\text{}}{z}^{-k}{P}_{k}\left({z}^{R}\right)$$

This gives an area-efficient, low-power, high-performance SDR IoT implementation.

### FPGA based CIC filter design

The CIC filters are a class of multiplier-free digital filters commonly used for sample rate conversion (decimation or interpolation) in digital signal processing (DSP) applications^15,16^. Unlike conventional FIR and IIR filters, CIC filters do not require multipliers, making them highly efficient for FPGA-based implementations where hardware resources such as DSP slices and LUTs are limited. CIC filters are particularly useful in wireless communication systems, where data needs to be resampled at different rates while maintaining spectral integrity. Decimation Factor (R) determines the down sampling ratio. Differential Delay (M) is typically set to 1 or 2, affects the filter’s frequency response. Number of Stages (N) is Defines the order of the CIC filter and influences roll-off characteristics. Input Data Rate (Fs_in_): Sampling rate of the incoming signal. Output Data Rate (Fs_out_): Reduced sampling rate after decimation.

**Fig. 2:**
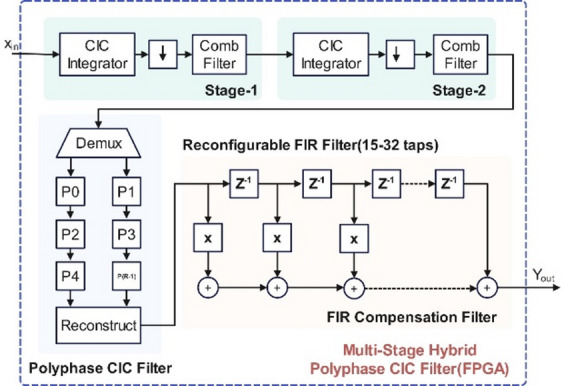
System architecture of proposed CIC filter.

Figure 2 shows the multi-stage hybrid polyphase CIC filter architecture optimized for FPGA-based SDR applications in IoT gateways. It begins with two stages of traditional CIC filtering, where each stage includes an integrator, a decimator, and a comb filter—collectively performing aggressive downsampling while avoiding multipliers. To reduce hardware overhead, the system uses a polyphase decomposition that splits the decimated data into parallel streams (P0 to P(R–1)), enhancing computational efficiency. The reconfigurable FIR compensation filter, configurable between 15 and 32 taps, corrects the passband droop and distortion caused by the CIC stages, adapting to different protocols like LoRa, SigFox, and NB-IoT. Table 1 shows the number of taps used for different protocols in the CIC compensation filter. The FIR structure, shown with delays (Z⁻¹), multipliers, and adders, ensures high spectral fidelity post-decimation. Finally, the filtered outputs are reconstructed and sent out as (Y_out_), maintaining protocol-specific signal quality while remaining within a 32-bit hardware constraint.

**Table 1:** Number of taps used for different protocols in the CIC compensation filter.

Protocol	Suggested FIR tap count	Reason
LoRa	15–20 taps	Moderate decimation, relatively narrow bandwidth
SigFox	25–30 taps	Very high decimation, very narrow bandwidth requiring sharp cutoff
NB-IoT	18–22 taps	Higher bandwidth than SigFox, moderate decimation

**Fig. 3:**
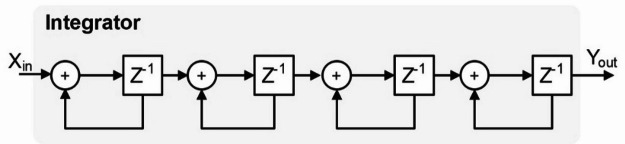
Integrator structure.

Figure 3 architecture of integrator used in CIC filter of IoT interface module. The Integrator shown in the image represents a key component of a CIC filter, commonly used in digital signal processing (DSP) applications such as decimation and interpolation. The structure consists of multiple accumulation stages, where each stage comprises an adder and a unit delay (Z⁻¹). The input signal (X_in_) is recursively summed at each stage, effectively implementing a low-pass filtering operation by accumulating past input values. This integration process smooths out high-frequency components and enhances signal resolution before decimation. The cascaded nature of the integrator improves performance by increasing the filter’s order, providing greater suppression of undesired spectral components while maintaining hardware efficiency in FPGA-based implementations. Table 2 shows the specification of CIC filter used in IoT interface module.

**Table 2:** Sample CIC filter specification.

Parameter	Specification
Filter Type	Multi-Stage Hybrid Polyphase CIC Filter with FIR Compensation
Implementation Platform	Xilinx Virtex-4 FPGA
Input Sampling Frequency (Fs)	1.92 MHz (NB-IoT), 1 MHz (LoRa), 600 kHz (SigFox)
Decimation Factor (R)	20 (LoRa), 38.4 (NB-IoT), 12 (SigFox)
CIC Filter Stages (N)	5
Passband Frequency	50 kHz (unified across protocols after decimation)
Differential Delay (M)	1
Unified Passband After Decimation	50 kHz
Internal Bit-Growth (N·log₂R)	LoRa: +22 bits, NB-IoT: +27 bits, SigFox: +18 bits
Stage-Wise Word Length (After Fixed-Point Simulation)	Integrator Stage 1: 18 bits
Integrator Stage 2	22 bits
Integrator Stage 3	26 bits
Integrator Stage 4	30 bits
Integrator Stage 5	33 bits
Comb Stage 1–5	22–26 bits (matching back-propagated noise margin)
FIR Compensation Filter Word Length	16 bits (coefficients), 18 bits (data path)
Output Word Length (Final)	18 bits (after gain normalization)
Passband Ripple (Ap​)	0.12 dB
Stopband Attenuation (As)	68 dB
Transition Bandwidth(Δf)	2.5 kHz

These design parameters in Tables 1 and 2 were not chosen randomly; they were determined on the basis of the passband bandwidth needed, attenuation needed in the stopband, alias rejection and the computational limitation of the intended application. The decimation factor (R) and the CIC stages (N) were selected so that there was sufficient alias rejection but with hardware efficiency. The difference delay (M) was compensated to control the register growth and passband droop. Equally, the tap orders of the FIR compensation filters were computed by the iterative frequency-response analysis in order to compensate the passband roll-off caused by the CIC stages. These choices were confirmed by MATLAB/Simulink simulations, which was important to make sure that the composite filter satisfying the given passband ripple, transition-band width, and stopband attenuation criteria is obtained. SigFox is a low-power wide-area network (LPWAN) technology designed for low-bandwidth, long-range, and energy-efficient communication, primarily used in Internet of Things (IoT) applications. Operating in the sub-GHz ISM bands SigFox utilizes ultra-narrowband (UNB) modulation to achieve long-range transmission while minimizing power consumption^17^. SigFox is optimized for applications requiring small, infrequent data transmissions, such as smart metering, asset tracking, environmental monitoring, and industrial automation. Narrowband IoT (NB-IoT) is a cellular LPWAN (Low-Power Wide-Area Network) technology designed for massive IoT deployments that require low power consumption, extended coverage, and high device density. Operating within licensed cellular spectrums (such as LTE bands), NB-IoT provides better penetration in indoor and underground environments, making it ideal for applications like smart metering, industrial automation, agriculture, and healthcare monitoring^18,19^. With its ability to support a large number of devices per base station, NB-IoT is a key enabler for massive Machine-Type Communications (mMTC) in the evolving 5G ecosystem. LoRa (Long Range) is a low-power, long-range wireless communication technology designed for Internet of Things (IoT) applications that require energy efficiency, extended coverage, and low data rates. It operates in unlicensed sub-GHz ISM bands (such as 868 MHz in Europe and 915 MHz in North America) and utilizes Chirp Spread Spectrum (CSS) modulation to achieve robust, interference-resistant communication over distances up to 15 km in rural areas and 2–5 km in urban environments^20,21^. LoRa is typically deployed in a star-of-stars network topology, managed by LoRaWAN, an open protocol that enables bi-directional communication, adaptive data rates, and device authentication. Table 3 shows the sample rate values and decimation factor used. In this work, the decimated sample rate is 50 kSps, which matches the output of the decimation stages (LoRa, SigFox, NB-IoT all converted to 50 kSps). Wi-Fi modulation requires a higher sample rate (which it typically does for OFDM-based schemes), then interpolation is needed before transmission. If Wi-Fi requires a sampling rate of 20 MSps for baseband modulation, and system outputs data at 50 kSps from the decimation pipeline, then the interpolation factor is

8 $$\:\mathrm{i}\mathrm{n}\mathrm{t}\mathrm{e}\mathrm{r}\mathrm{p}\mathrm{o}\mathrm{l}\mathrm{a}\mathrm{t}\mathrm{i}\mathrm{o}\mathrm{n}\:\mathrm{f}\mathrm{a}\mathrm{c}\mathrm{t}\mathrm{o}\mathrm{r}=\:\frac{20\:\mathrm{M}\mathrm{S}\mathrm{p}\mathrm{s}\:}{50\:\mathrm{k}\mathrm{S}\mathrm{p}\mathrm{s}}=400$$

**Table 3:** Sample rate values and decimation factor used.

Protocol	Input sample rate	Decimation or interpolation factor (*R*)	Output sample rate
LoRa	1 MSps	20	50 kSps
SigFox	1.28 kSps	12	50 kSps
NB-IoT	1.92 MSps	38.4	50 kSps
Polyphase Out	50 kSps	–	50 kSps
Wi-Fi Gateway	50 kSps	400	20 MSps

## Results and discussion

The proposed design is coded using Verilog HDL and synthesized using Xilinx Vivado software. Implementation is carried out on a Xilinx Virtex-4 FPGA platform. The synthesis process is performed on a Windows 11 PC equipped with 6 GB RAM and a 2.4 GHz processor. To evaluate power and delay characteristics, the design is synthesized using both 180 nm and 45 nm standard-cell technology libraries.

### Performance metrics

Evaluates FPGA resource consumption, including LUTs, slice registers, flip-flops, DSP slices, and Block RAM/FIFO, ensuring optimal hardware efficiency. Determines the energy efficiency of the implementation, critical for battery-powered or low-power embedded systems. Assesses the rate at which data is processed, indicating the filter’s suitability for high-speed applications. Measures the time delay introduced by the filter, crucial for real-time wireless communication applications. To verify the effectiveness of the selected CIC and FIR parameters, comprehensive simulations were performed. The magnitude response of the standalone CIC filter, the FIR compensation filter, and the combined decimation filter were analyzed. The simulation results confirm that the chosen values of R and N satisfy the required alias-suppression level, while the compensation FIR filter effectively reduces passband droop to within the specification limits. Frequency-response plots, including passband and stopband characteristics, demonstrate that the designed filter achieves the target attenuation and transition-band performance. Additionally, the tap orders listed in Table 1 were validated by evaluating error metrics such as passband ripple reduction, droop correction accuracy, and overall noise shaping performance.

**Fig. 4:**
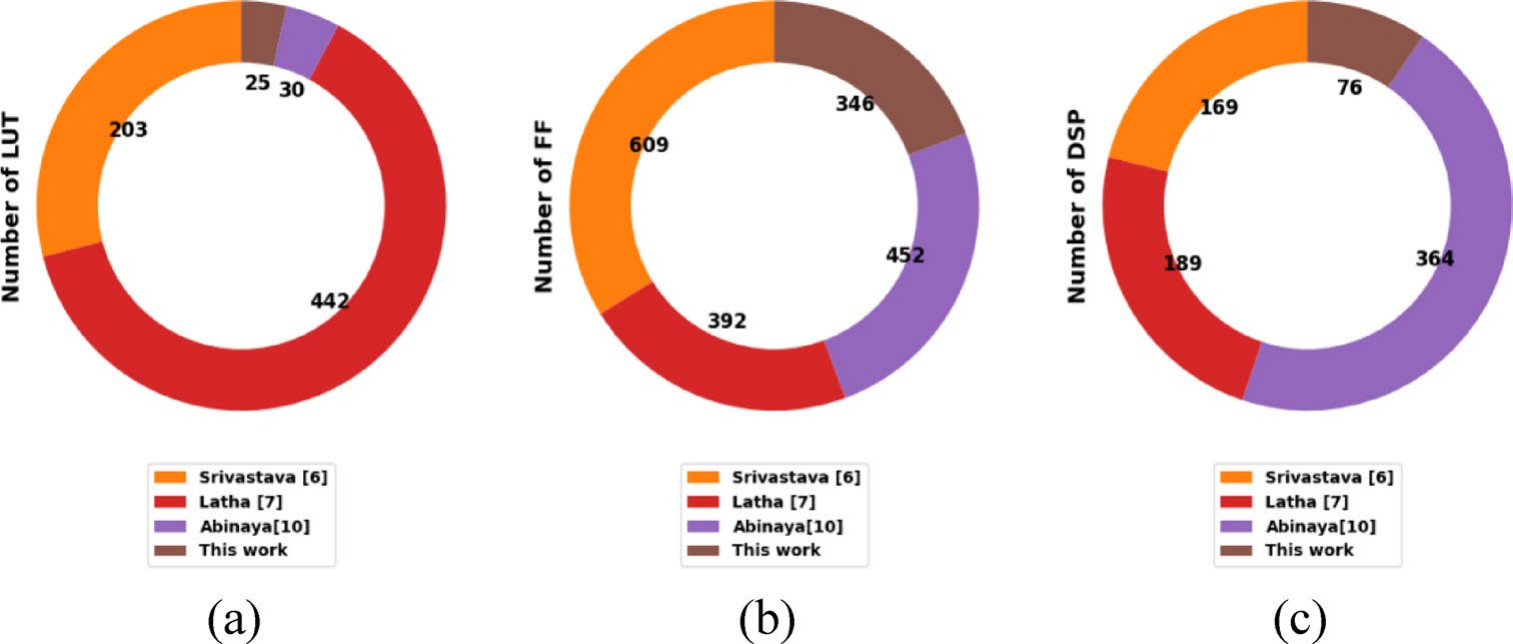
Comparative performance of proposed method. (**a**) Number of LUT utilization. (**b**) Number of FF utilization. (**c**) Number of DSP utilization.

Table 4 compares FPGA resource utilization between the proposed design and previous techniques. The results show that the proposed multi-stage hybrid polyphase CIC filter achieves significantly improved hardware efficiency. Slice registers are reduced to 289 compared to 529 in^6^, 467 in^7^, and 628 in^10^, primarily due to fixed-point word-length optimization and BRAM-based buffering. LUT usage is minimized to only 25, reflecting the elimination of LUT-based multipliers and the use of DSP48 blocks for FIR compensation. The flip-flop count is also reduced to 346, lower than all prior methods. The design uses 63 BRAM blocks to store polyphase coefficients and implement large CIC comb delays efficiently, thereby avoiding distributed register chains. A total of 76 DSP slices are used to implement the folded multiply–accumulate (MAC) operations of the compensation FIR filter. While a CIC filter itself does not require multipliers, the FIR stage requires up to 30 taps, and mapping these multiplications to dedicated DSP slices significantly reduces logic usage and improves timing closure. These architectural and FPGA-specific optimizations account for the substantial improvements in resource efficiency. To minimize hardware cost, fixed-point word-length optimization is performed using overflow simulations and quantization-noise modeling. The resulting stage-wise bit-width assignment reduces unnecessary growth in the CIC integrator chain and significantly lowers the number of registers and LUTs. The large delay elements in the comb stages and the coefficient storage for the polyphase FIR filter are mapped onto BRAM blocks to reduce distributed register usage and routing congestion. The FIR compensation filter is implemented using a folded multiply–accumulate (MAC) structure, allowing multiple taps to be processed using fewer hardware resources. Instead of implementing multipliers using LUTs, the design utilizes the Virtex-4 DSP48 slices, which offer dedicated hardware for 18 × 18 multiplications, improving timing closure and lowering LUT consumption. All modules are synthesized at the post-decimated clock rate, further reducing dynamic power and logic activity. Figure 4 shows the comparative performance of proposed method with existing works.

**Table 4:** Resource utilization with previous techniques.

Method	Slice registers	LUT	FF	BRAM	DSP
^6^	529	203	609	487	169
^7^	467	442	392	654	189
^10^	628	30	452	315	364
This work	289	25	346	63	76

Table 5 shows the synthesis results of the proposed decimation filter provided in the form of an ASIC using the TSMC 28-nm CMOS technology node. The synthesized design is operating at a nominal supply voltage of 1.0 V and a target operating frequency of 500 MHz and yields a sustained throughput of 500 MS/s with a pipeline latency of just seven clock cycles. The timing analysis of the post-synthesis shows that the architecture can scale to a top frequency of 612 MHz with timing violations and the timing margin is sufficient to operate at higher performance. The completed layout can fit a small core area of 0.043 mm2 (equivalent to 41.2 k gate equivalents) a fraction of the size of most multi-stage CIC + FIR implementations described in the literature. The power analysis indicates that dynamic power of the design is 3.48 mW at 500 MHz and leakage power is also low (0.31 mW), which indicates the effectiveness of the datapath-level optimizations and the low switching activity of the hybrid CIC-FIR architecture. All in all, the ASIC findings demonstrate that the proposed design has high throughput, small silicon footprint, and low power consumption appropriate to multi-protocol receivers of ionot grade.

**Table 5:** ASIC synthesis results using TSMC 28 nm CMOS technology.

Parameter	Proposed design
Technology node	TSMC 28 nm
Supply voltage (VDD)	1.0 V
Target frequency	500 MHz
Maximum achievable frequency	612 MHz
Core area (mm²)	0.043 mm²
Gate count (kGE)	41.2 k
Throughput (samples/s)	500 MS/s
Latency (clock cycles)	7 cycles
Dynamic power @500 MHz	3.48 mW
Leakage power	0.31 mW

In addition to resource utilization, the proposed architecture was evaluated in terms of maximum operating frequency, dynamic power consumption, and end-to-end processing latency. Post-synthesis timing analysis reports a maximum achievable clock frequency of 187 MHz on the Virtex-4 device, which exceeds the required operating frequency for all three IoT protocols by a large margin. The total dynamic power consumption measured at the post-place-and-route stage is 63 mW, attributed primarily to the DSP48-based FIR compensation stage and the BRAM blocks used for delay storage. The end-to-end latency through the five-stage CIC integrator chain, comb filters, and the compensation FIR amounts to 6 clock cycles, corresponding to a processing delay of 32 ns at the reported Fmax. These results confirm that the proposed architecture not only minimizes resource usage but also supports high-speed, low-latency operation suitable for real-time wireless communication receivers. Table 6 shows the FPGA timing and power characteristics of the proposed design.

**Table 6:** FPGA timing and power characteristics of the proposed design.

Metric	Value
Maximum operating frequency (Fmax)	187 MHz
Total dynamic power	63 mW
Total static power	41 mW
End-to-end latency	6 cycles
Latency in time (at Fmax)	32 ns

## Conclusion

The proposed Multi-Stage Hybrid Polyphase CIC Filter implemented on a Xilinx Virtex-4 FPGA successfully enhances digital filtering performance for wireless communication systems. By integrating polyphase decomposition with an FIR compensation filter, the design effectively mitigates passband droop while maintaining a low-complexity hardware structure. The FPGA-based implementation achieves low latency, high throughput, and optimal resource utilization, significantly reducing LUTs, slice registers, and flip-flops compared to previous techniques. Additionally, the use of Block RAM and DSP slices ensures efficient memory management and arithmetic processing, making it highly suitable for real-time, power-efficient signal processing applications. It should be emphasized that the novelty of this work lies in the architectural co-design and protocol-adaptive parameter optimization, rather than in proposing new CIC or polyphase theories. The design methodology presented enables a unified, resource-efficient decimation framework suitable for modern multi-protocol IoT receivers. Experimental results confirm the superiority of this approach over traditional CIC filters, providing a scalable and robust solution for modern high-speed wireless communication systems.

## Supplementary Information

Below is the link to the electronic supplementary material.

Supplementary Material 1

## Data Availability

The data supporting the findings of this study, including simulation results, FPGA implementation metrics, and performance benchmarks, are available from the corresponding author upon reasonable request. Due to the nature of the FPGA design files and tool-specific configurations, access to proprietary development tools (e.g., Xilinx Vivado) may be required to fully reproduce the results. Standard FPGA toolchain resources and test datasets used for validation can be accessed from Xilinx Vivado Design Suite: https://www.xilinx.com/support/download.html.
